# The Use of Baclofen as a Treatment for Alcohol Use Disorder: A Clinical Practice Perspective

**DOI:** 10.3389/fpsyt.2018.00708

**Published:** 2019-01-04

**Authors:** Renaud de Beaurepaire, Julia M. A. Sinclair, Mathis Heydtmann, Giovanni Addolorato, Henri-Jean Aubin, Esther M. Beraha, Fabio Caputo, Jonathan D. Chick, Patrick de La Selle, Nicolas Franchitto, James C. Garbutt, Paul S. Haber, Philippe Jaury, Anne R. Lingford-Hughes, Kirsten C. Morley, Christian A. Müller, Lynn Owens, Adam Pastor, Louise M. Paterson, Fanny Pélissier, Benjamin Rolland, Amanda Stafford, Andrew Thompson, Wim van den Brink, Lorenzo Leggio, Roberta Agabio

**Affiliations:** ^1^Groupe Hospitalier Paul-Guiraud, Villejuif, France; ^2^Faculty of Medicine, University of Southampton, Southampton, United Kingdom; ^3^Department of Gastroenterology, Royal Alexandra Hospital Paisley, Paisley, United Kingdom; ^4^AUD and Alcohol Related Diseases Unit, Department of Internal Medicine and Gastroenterology, Fondazione Policlinico Universitario A Gemelli Istituto di Ricovero e Cura a Carattere Scientifico, Rome, Italy; ^5^Department of Internal Medicine and Gastroenterology, Università Cattolica del Sacro Cuore, Rome, Italy; ^6^Faculté de Médecine, Centre de Recherche en Epidémiologie et Santé des Populations, Université Paris-Sud, Paris, France; ^7^Faculté de Médecine, Université de Versailles Saint-Quentin-en-Yvelines, Paris, France; ^8^Institut National de la Santé et de la Recherche Médicale, Université Paris-Saclay, Paris, France; ^9^Hôpitaux Universitaires Paris-Sud, Paris, France; ^10^Department of Psychology, University of Amsterdam, Amsterdam, Netherlands; ^11^Department of Internal Medicine, SS. Annunziata Hospital, Cento, Italy; ^12^Castle Craig Hospital, Blyth Bridge, United Kingdom; ^13^School of Health and Social Care, Edinburgh Napier University, Edinburgh, United Kingdom; ^14^Private Practice, Montpellier, France; ^15^Department of Addiction Medicine, Poisons and Substance Abuse Treatment Centre, Toulouse-Purpan University Hospital, Toulouse, France; ^16^Department of Psychiatry, School of Medicine, University of North Carolina at Chapel Hill, Chapel Hill, NC, United States; ^17^National Health Medical Research Council, Centre of Research Excellence in Mental Health and Substance Use, Central Clinical School, Sydney Medical School, University of Sydney, Sydney, NSW, Australia; ^18^Drug Health Services, Royal Prince Alfred Hospital, Camperdown, NSW, Australia; ^19^Département de Médecine Générale, Faculté de Médecine, Université Paris Descartes, Paris, France; ^20^Neuropsychopharmacology Unit, Division of Brain Sciences, Centre for Psychiatry, Imperial College London, London, United Kingdom; ^21^Discipline of Addiction Medicine, Faculty of Medicine and Health, University of Sydney, Sydney, NSW, Australia; ^22^Department of Psychiatry, Campus Charité Mitte, Charité-Universitätsmedizin Berlin, Berlin, Germany; ^23^Wolfson Centre for Personalised Medicine, University of Liverpool, Liverpool, United Kingdom; ^24^Department Addiction Medicine, St Vincent's Hospital Melbourne, Melbourne, VIC, Australia; ^25^Department of Medicine, University of Melbourne, Melbourne, VIC, Australia; ^26^Poison Control Center, Toulouse-Purpan University Hospital, Toulouse, France; ^27^Service Universitaire d'Addictologie de Lyon, Lyon, France; ^28^University of Lyon, Lyon, France; ^29^Royal Perth Hospital, Perth, WA, Australia; ^30^Department of Psychiatry, Amsterdam University Medical Centers, Academic Medical Center, Amsterdam University, Amsterdam, Netherlands; ^31^Section on Clinical Psychoneuroendocrinology and Neuropsychopharmacology, Division of Intramural Clinical and Basic Research, National Institute on Alcohol Abuse and Alcoholism, National Institute on Drug Abuse Intramural Research Program, National Institutes of Health, Bethesda, MD, United States; ^32^Medication Development Program, National Institute on Drug Abuse Intramural Research Program, National Institutes of Health, Baltimore, MD, United States; ^33^Department of Behavioral and Social Sciences, Center for Alcohol and Addiction Studies, Brown University, Providence, RI, United States; ^34^Section of Neuroscience and Clinical Pharmacology, Department of Biomedical Sciences, University of Cagliari, Cagliari, Italy

**Keywords:** GABA-B, baclofen, alcohol use disorder, efficacy, safety

## Abstract

Alcohol use disorder (AUD) is a brain disorder associated with high rates of mortality and morbidity worldwide. Baclofen, a selective gamma-aminobutyric acid-B (GABA-B) receptor agonist, has emerged as a promising drug for AUD. The use of this drug remains controversial, in part due to uncertainty regarding dosing and efficacy, alongside concerns about safety. To date there have been 15 randomized controlled trials (RCTs) investigating the use of baclofen in AUD; three using doses over 100 mg/day. Two additional RCTs have been completed but have not yet been published. Most trials used fixed dosing of 30–80 mg/day. The other approach involved titration until the desired clinical effect was achieved, or unwanted effects emerged. The maintenance dose varies widely from 30 to more than 300 mg/day. Baclofen may be particularly advantageous in those with liver disease, due to its limited hepatic metabolism and safe profile in this population. Patients should be informed that the use of baclofen for AUD is as an “off-label” prescription, that no optimal fixed dose has been established, and that existing clinical evidence on efficacy is inconsistent. Baclofen therapy requires careful medical monitoring due to safety considerations, particularly at higher doses and in those with comorbid physical and/or psychiatric conditions. Baclofen is mostly used in some European countries and Australia, and in particular, for patients who have not benefitted from the currently used and approved medications for AUD.

## Introduction

After promising preclinical evidence [for review see Colombo and Gessa (1)], clinical studies started to investigate whether baclofen may be useful in the treatment of alcohol use disorder (AUD). However, to date, clinical studies have yielded conflicting results. Despite the lack of consistent evidence, baclofen is often used off-label in clinical practice to treat AUD, especially in some European countries and Australia (2). In this manuscript, a large group of researchers and clinicians combine their expertise in this area to provide (a) a review of the current research evidence and clinical experience of using baclofen in the treatment of AUD, (b) a description of the two different approaches used to administer baclofen in clinical practice settings (“fixed doses” or “flexible doses”) to treat AUD, and (c) a brief overview of the clinical use of baclofen to treat AUD.

## Review Of The Current Research Evidence And Clinical Experience

### Alcohol Use Disorder and the Need for Additional Medications

AUD is a major public health problem associated with high rates of mortality and morbidity worldwide (3–7). The previous editions of the Diagnostic and Statistical Manual of Mental Disorders (DSM) described two disorders related to a pattern of maladaptive alcohol consumption, alcohol abuse and alcohol dependence [e.g., DSM-IV; 8]. The diagnosis of dependence required the fulfillment of three (or more) criteria out of a set of seven, whereas the diagnosis of abuse required at least one out of four different criteria. These two disorders have been combined into a single disorder (AUD), where one set of criteria is now used (DSM-5; 3). In DSM-5, the AUD diagnosis requires the fulfillment of two (or more) criteria out of a set of 11, including “craving,” or a strong desire or urge to use alcohol (3), instead of legal problems (included among DSM-IV alcohol abuse criteria, but excluded by DSM-5 AUD criteria). Accordingly, the previous diagnosis (DSM-IV) of alcohol dependence corresponds approximately to moderate/severe DSM-5 AUD (9).

AUD is characterized by periods of excessive alcohol consumption and a chronic relapsing, remitting course (3). According to the different AUD phases, the goal of medical treatment may be to achieve and maintain abstinence, if patients are currently drinking—or just maintain abstinence.

Even if abstinence is the best goal for AUD medical treatment, some patients prefer to reduce their alcohol consumption to low-risk drinking, instead of total abstinence. According to the US National Institute on Alcohol Abuse and Alcoholism (NIAAA), low-risk drinking corresponds to an alcohol consumption ≤ 14 (men) or 7 (women) drinks per week and ≤ 5 (men) or 4 (women) drinks on a single day (1 drink = 14 g alcohol) (10). This consumption pattern is associated with lower risks than moderate/high risk drinking, even if the safest level of drinking is none (5).

Accordingly, the recent guidelines of the American Psychiatric Association for the pharmacological treatment of AUD recommend the use of naltrexone, acamprosate, or disulfiram, based on the treatment objective (reducing alcohol consumption or achieving and maintaining abstinence), patient preference, and presence of comorbidities that may contraindicate a specific drug (11). In Europe, nalmefene has also been approved for the treatment of alcohol dependence (12). Nevertheless, only a minority of people with AUD seek and receive medical treatment (4, 13, 14) and current approved medications for AUD are of limited effectiveness (15). Therefore, identification of other medications may contribute toward increasing the number of AUD patients who benefit from pharmacological treatments with a different mechanism of action.

### Baclofen and Alcohol Use Disorder

Baclofen, a selective gamma-aminobutyric acid-B (GABA-B) receptor agonist, has emerged as a promising drug for AUD (16). It has been marketed since the early 1970s for the treatment of muscle spasticity, secondary to neurological conditions. The wide use of baclofen as a myorelaxant has provided detailed information on its safety and side effects in these patients (17). From the 1970s, research, largely in animal addiction models, suggested that baclofen may also be effective in the treatment of AUD (1).

## Evidence For the Effect of Baclofen on Alcohol Use

### Preclinical Studies

Animal studies showed that baclofen induced a dose-related reduction in (a) the behavioral effects caused by alcohol (18), (b) acquisition and maintenance of alcohol consumption (19–21), including binge-like drinking (22), (c) relapse-like drinking (23), (d) severity of alcohol withdrawal signs (20), (e) cue-induced reinstatement of previously extinguished alcohol seeking behavior (24), and (f) reinforcing and motivational properties of alcohol (25–30) in different validated rodent models of AUD [for a recent review, see Colombo and Gessa (1)].

### Clinical Studies

#### Studies Using baclofen 30 mg/Day

Addolorato and colleagues were the first to investigate the efficacy of baclofen in reducing alcohol consumption in AUD patients (31). In this first study, 10 male AUD patients received 30 mg/day baclofen (starting from 5 mg, three times a day, and then 10 mg, three times a day) for four consecutive weeks. Patients reported their last alcohol intake in the preceding 24 h. Seven patients achieved and maintained abstinence, and another two significantly reduced their alcohol consumption. Flannery and colleagues replicated these findings in 12 AUD patients, including three women, that were active drinkers (three days abstinent before the beginning of the trial), using the same dose of baclofen for 12 weeks (32). However, conclusions that can be drawn from these results are limited by the open-label design and the absence of a (placebo) control group.

In 2002, Addolorato and colleagues conducted the first randomized controlled trial (RCT) (see Table 1). In this RCT, 39 AUD male participants received 30 mg/day baclofen or placebo, for 4 weeks. Participants were active drinkers (last intake in the preceding 24 h), did not suffer from any other mental disorder, were treated as outpatients, and received psychological support every week. Their mean baseline alcohol consumption was 17.6 drinks per day (1 drink = 12 g of alcohol) in the baclofen group. Compared to placebo, baclofen increased the percentage of patients who achieved and maintained abstinence (abstinent patients), as well as the number of abstinent days, and decreased the number of drinks per drinking day and anxiety levels (33).

**Table 1:** Randomized double-blind placebo-controlled trials.

**References**	**Daily dose of baclofen; Number of participants**	**Mean of drinks per drinking days**	**Weeks of duration**	**Significant difference between baclofen and placebo**	**Effects of baclofen compared to placebo**
Addolorato et al. (33)	BAC (30 mg): 20 PLA: 19	BAC (30 mg): 18.0^a^ PLA: 10.0	4	Yes	BAC (30 mg): ↓ 100.0% DD PLA: ↓ 60.0% DD
Garbutt et al. (34)	BAC (30 mg): 40 PLA: 40	BAC (30 mg): 7.3^b^ PLA: 6.9	12	No	BAC (30 mg): 51.7% abstinent days PLA: 51.6% abstinent days
Addolorato et al. (35)	BAC (30 mg): 42 PLA: 42	BAC (30 mg): N.A. PLA: N.A.	12	Yes	BAC (30 mg): 71.4% abstinent patients PLA: 28.6% abstinent patients
Hauser et al. (36)	BAC (30 mg): 88 PLA: 92	BAC (30 mg): 7.1^b^ PLA: 7.6	12	No	BAC (30 mg): 32.3% abstinent days PLA: 31.1% abstinent days
Ponizovsky et al. (37)	BAC (50 mg): 32 PLA: 32	BAC (50 mg): 7.4^a^ PLA: 8.2	12	No	BAC (50 mg): 46.1% abstinent days PLA: 47.5% abstinent days
Krupitsky et al. (38)	BAC (50 mg): 16 PLA: 16	BAC (50 mg): 0.1^a^ PLA: 0.3	12	No	BAC (50 mg): 100% abstinent days last week PLA: 100% abstinent days last week
Addolorato et al. (39)	BAC (30 mg): 14 BAC (60 mg): 14 PLA: 14	BAC (30 mg): 13.9^a^ BAC (60 mg): 9.6 PLA: 12.0	12	Yes	BAC (30 mg): ↓ 53% DD BAC (60 mg): ↓ 68% DD PLA: N.A.
Morley et al. (40)	BAC (30 mg): 14 BAC (60 mg): 14 PLA: 14	BAC (30 mg): 15.5^c^ BAC (60 mg): 15.1 PLA: 14.3	12	No	BAC (30 mg): 5.9 DDD BAC (60 mg): 5.6 DDD PLA: 2.8 DDD BAC (30 mg) induced positive effects among patients with comorbid anxiety
Morley et al. (41)	BAC (30 mg): 36 BAC (75 mg): 35 PLA: 33	BAC (30 mg): 17.0^c^ BAC (75 mg): 13.8 PLA: 14.1	12	Yes	BAC (30 mg): 68.5% abstinent days BAC (75 mg): 64.6% abstinent days PLA: 43.3% abstinent days
Leggio et al. (42)	BAC (80 mg): 15 PLA: 15	BAC (80 mg): N.A. PLA: N.A.	12	Yes	BAC (80 mg): 12.1% abstinent days from alcohol and tobacco PLA: 3.5% abstinent days from alcohol and tobacco
Garbutt et al. unpublished	BAC (30 mg) BAC (90 mg) PLA	-	-	-	-
Beraha et al. (43)	BAC (30 mg): 31 BAC (up to 150 mg): 58 PLA: 62	BAC (30 mg): 11.0^a^ BAC (up to 150 mg): 12.2 PLA: 11.8	16	No	BAC (30 mg): 41.9% abstinent patients BAC (up to 150 mg): 43.1% abstinent patients PLA: 46.8% abstinent patients
Reynaud et al. (44)	BAC (up to 180 mg): 155 PLA: 155	BAC (up to 180 mg): 8.0^a^ PLA: 7.8	26	No	BAC (up to 180 mg): 11.9% abstinent patients PLA: 10.5% abstinent patients
Müller et al. (45)	BAC (up to 270 mg): 28 PLA: 28	BAC (up to 270 mg): 17.2^a^ PLA: 16.0	12	Yes	BAC (up to 270 mg): 68.2% abstinent patients PLA: 23.8% abstinent patients
Jaury et al., unpublished	BAC (up to 300 mg) PLA	-	-	***-***	-

a *1 drink = 12 g of alcohol*;

b 1 drink = 14 g of alcohol

c *1 drink = 10 g of alcohol; BAC, baclofen; DD, drinking days; DDD, drinks per drinking days; N.A., not applicable; PLA, Placebo*.

However, a similar RCT found different results [(34); see Table 1]. In this RCT, 80 AUD patients, active drinkers (three days abstinent before beginning the trial), received either 30 mg/day baclofen or placebo for 12 weeks, in an outpatient setting, together with eight sessions of a comprehensive psychological intervention named BRENDA. In this study, there was no difference between baclofen and placebo in the percentage of heavy drinking days, abstinent days, time to first drink (time to lapse), or time to heavy drinking day (time to relapse).

This RCT differed from the Addolorato et al. (33) in several aspects: (a) the high number of women recruited (45% females); (b) the number of individuals who suffered from other mental disorders (e.g., 29% on antidepressants); (c) the low amount of alcohol consumed at baseline (7.3 drinks per day with 1 drink = 14 g of alcohol in baclofen group); (d) the low baseline levels of alcohol withdrawal; (f) the high placebo response; (g) the different aims of the treatment (including abstinence, occasional use, and regular but reduced use); and (h) financial compensation for attending each visit (46).

More recently, three RCTs (each with three arms) compared the efficacy of 30 mg/day (10 mg, three times a day) to another dose of baclofen and placebo in the treatment of AUD, for 12 weeks (39–41).

Regarding participants treated with 30 mg/day baclofen compared to placebo, the first RCT found that baclofen significantly reduced the number of drinks per drinking day (39), whereas the second RCT found no difference in time to relapse nor time to lapse (40). The third RCT found that baclofen treatment (both dose group composite), compared to placebo, increased (a) time to first lapse, (b) time to first relapse, and (c) percentage of days abstinent. The characteristics of these RCTs are described in detail below. Recently, another 3-arm RCT (30 mg/day, 90 mg/day and placebo) was completed, and data analysis is underway (Garbutt JC, unpublished; https://clinicaltrials.gov/, identifier NCT01980706).

Two additional RCTs tested baclofen 30 mg/day in AUD patients with liver disease. In 2007, Addolorato et al. investigated the efficacy of baclofen in AUD patients with liver cirrhosis (Table 1). The rationale for selecting this specific sample was that, in these patients, certain AUD pharmacological agents (e.g., disulfiram and naltrexone) are contraindicated because of their liver metabolism, whereas baclofen has lower liver metabolism and primarily renal excretion. In this RCT, 42 outpatients received 30 mg/day baclofen and 42 received placebo for 12 weeks (35). Participants were active drinkers [at least two heavy drinking days per week and an average consumption of 21/14 drinks (men/women) per week, or more, during the 4 weeks before enrollment], included 23 women (27.4% of the entire sample), did not suffer from other severe mental disorders, and were seen every week for the first month, and then every 2 weeks. At each visit, patients received an individual session of counseling support lasting 30 min. At the end of the 12-weeks study, a higher rate of participants allocated baclofen achieved and maintained abstinence and had a longer cumulative abstinence duration compared with placebo.

More recently, Hauser et al. (36) conducted a similar RCT among AUD patients with chronic hepatitis C (HCV), enrolled at four US Veteran Affairs Medical Centers (Table 1), and found that 30 mg/day baclofen did not increase abstinence or reduce alcohol use, craving for alcohol, or anxiety compared to placebo. In this RCT, 40 participants received 30 mg/baclofen and 40 participants placebo for 12 weeks. These patients were active drinkers (at least one heavy drinking day per week or more than 7 drinks per week, for each of the preceding 2 weeks), did not suffer from other mental disorders, and were seen every week for the first month, and then every 2 weeks. However, unlike the RCT by Addolorato et al. (35), the Hauser et al. study (36) included only three women (3.7% of the entire sample), had low baseline levels of alcohol consumption (7.1 drinks per drinking days with 1 drink = 14 g of alcohol in baclofen group), and participants received manual-guided counseling lasting 15 min at each visit.

#### Anecdotal and Open-Label Observations With Doses of baclofen >30 mg/Day

Some case reports (47–49) suggested the potential utility of increasing the doses of baclofen to treat patients with AUD. The first of these case reports was published in 2005 by Olivier Ameisen, a physician suffering from severe AUD, who reported that he achieved abstinence from alcohol with 270 mg/day of baclofen (47). Similar observational studies started being published from 2010 (50–62). These case-series (without control groups) suggested that doses of baclofen ranging from 30 to more than 300 mg/day may be effective, with some patients achieving up to a maximum daily dose of 400 mg (55, 63). The effectiveness of baclofen was also reported in AUD patients affected by liver disease (55, 64, 65).

#### RCTs Using Doses of Baclofen >30 mg/day and <100 mg/Day

Subsequently, a series of RCTs investigated the efficacy of higher doses of baclofen in the treatment of AUD compared to those administered in early studies (Table 1), as detailed below.

Two RCTs investigated the efficacy of 50 mg/day baclofen administered in two, instead of three, times a day [37, 38, Table 1]. In both studies, participants did not suffer from other severe mental disorders, were seen every week, as outpatients, for 12 weeks, and received an individual psychosocial intervention. In one RCT, 64 AUD participants included 16 women (25% of total sample), consumed 7.4 drinks per drinking day (patients allocated to baclofen treatment; 1 drink = 12 g of alcohol), and, other than the weekly individual intervention, also received group counseling sessions, every 2 weeks (37). Participants were active drinkers (at least two heavy drinking days per week and average overall consumption >21/14 drinks per week (men/women) during the month preceding recruitment, and no more than six abstinent days per month). This study did not find differences in the percentages of heavy drinking and abstinent days between the baclofen and the placebo group. However, a high placebo effect was observed (e.g., percentage of abstinent days was 47.5% for placebo and 46.1% for baclofen).

In the second RCT, 32 AUD participants were abstinent from alcohol consumption for at least 7 days and consumed 8.5 g of pure ethanol per week at baseline (for patients allocated to baclofen treatment, equal to ~0.1 drink per drinking day if patients drink every day, 1 drink = 12 g of alcohol) and the number of women recruited was not provided (38). The study found no differences between baclofen and placebo in alcohol consumption and time to relapse.

Two RCTs (each with three arms) compared the efficacy of 60 mg/day (20 mg three times a day) to 30 mg/day baclofen and placebo in the treatment of AUD, for 12 weeks (39, 40). The first RCT found that, compared with patients allocated to placebo, participants treated with baclofen significantly reduced the number of drinks per drinking day and this effect was greater among participants treated with 60 mg/day baclofen than those with 30 mg/day (39). Participants included 32 men (76%) and 10 women (24%) and did not suffer from other severe mental disorders. They were active drinkers (at least two heavy drinking days per week and an average overall consumption of >21/14 drinks (men/women) per week during the 4 weeks before enrollment, and ability to refrain from drinking at least 3 days before randomization day) and consumed a mean of ~12 drinks per drinking day at baseline, with 1 drink = 12 g of alcohol). Each participant was seen as an outpatient, every week for the first month, and then every 2 weeks. At each visit, patients received an individual session of counseling support lasting 30 min.

In contrast, the second RCT found no difference between baclofen 60 mg, baclofen 30 mg, and placebo on time to relapse, nor time to lapse (40). Participants included 19 men (45%) and 23 women (55%), were active drinkers (abstinent from alcohol at least 3 days prior to randomization) and consumed high levels of alcohol at baseline [more than 15 drinks per drinking day (1 drink = 10 g of alcohol) in baclofen groups], were seen as outpatients every week for the first month, then every 2 weeks, and, at each visit, received 30-min psychosocial therapy. In addition, 41% of participants suffered with current anxiety disorders. A *post-hoc* analysis showed a beneficial effect of baclofen, compared to placebo, only among AUD patients with comorbid anxiety disorders. Namely, AUD patients with anxiety disorder treated with baclofen had the first lapse and relapse after a significantly longer period of time, compared to AUD patients with anxiety treated with placebo. However, no difference was found between 60 and 30 mg/day baclofen.

One RCT compared the efficacy of 75 mg/day (25 mg three times a day) to 30 mg/day baclofen and placebo in the treatment of 104 AUD patients (including 30 (29%) women), for 12 weeks [(41); Table 1]. In this study, participants were seen as outpatients every 2 weeks, and, at each visit, received adherence therapy lasting 20–60 min. People with active major mental disorders were excluded, but 55% of participants were prescribed antidepressants and 56% suffered from liver disease (with or without cirrhosis). Participants were abstinent from alcohol consumption for between three and 21 days and their baseline level of alcohol consumption was equal to 15 drinks per drinking day with 1 drink = 10 g of alcohol). The aims of treatment included both abstinence and reduction of alcohol consumption. The study found that baclofen treatment (both dose groups combined), compared to placebo, increased: (a) time to first lapse, (b) time to first relapse, and (c) percentage of days abstinent. However, there were no differences between the effects of the 75 mg/day and the 30 mg/day groups. When the results were analyzed according to the presence of liver disease, baclofen (both dose group composite) was shown to be effective in increasing the time to lapse and relapse among participants affected by liver disease, but not among those without liver disease.

One RCT investigated the efficacy of 80 mg/day (20 mg at 4 times/day) baclofen in the treatment of 30 patients affected by AUD and nicotine use disorder [(42); Table 1]. In this 12-weeks study, consistent with FDA recommendations, the daily dose of baclofen 80 mg/day was divided into four administrations (20 mg, four times a day). Participants included 12 females (40%), did not suffer from other severe mental disorders, were seen as outpatients every week for the first month, then every 2 weeks, and, at each visit, received an individual session of medical management. They were active smokers and drinkers at the beginning of the trial and their consumption of alcohol at baseline was high but expressed as percent of heavy drinking days (78% for patients allocated to baclofen). Participants were looking for treatment for both AUD and smoking, but with different treatment goals (i.e., reducing or quitting both substances or quitting one and reducing the other). Regarding alcohol consumption, 48% of overall participants wanted to quit drinking. The results of the study showed that the rate of abstinent days from co-use of alcohol and tobacco was higher among participants treated with baclofen compared to those treated with placebo (42).

#### RCTs Using Doses of Baclofen >100 mg/Day

A recent RCT compared the efficacy of an intended maximum dose of 150 mg/day baclofen (in three daily administrations) to 30 mg/day baclofen and placebo in 151 AUD patients [(43); Table 1]. The trial did not find any difference between the three groups in any outcome evaluated (time to first relapse, total alcohol consumption, and proportion of abstinent patients). However, the results showed a very high placebo effect (e.g., 66% of participants allocated to placebo remained abstinent for the full study period). This study also included patients (31% females) with comorbid depression, anxiety, and bipolar disorder. Participants were abstinent for a mean of ~12 days (range: 4–21 days) and their baseline levels of alcohol consumption were equal to 141.8 g per day (equal to ~12 drinks per drinking day when 1 drink = 12 g of alcohol). The RCT comprised two phases. In the first phase (lasting 6 weeks), participants gradually increased the daily dose of baclofen up to 150 mg depending on tolerance (titration phase; 10 mg every other day, up to 30 mg/week). In the second phase (lasting 10 weeks), participants received the maximum dose achieved during the previous phase. Participants in both baclofen groups started with 30 mg/day (in three daily administrations) from the first day of treatment. Participants allocated to baclofen 30 mg/day received the same dose for the whole study period (16 weeks). In this multicenter trial, the setting varied between the centers. The majority of participants were treated as inpatients for the first 4–6 weeks (79%) followed by 10–12 weeks outpatient treatment. In all centers, participants received weekly group or individual psychotherapy sessions. The results of the trial showed that among participants allocated to baclofen, up to 150 mg/day, only 16% achieved the highest dose. Overall, these patients received a mean of 93.6 mg/day baclofen.

Another multicenter RCT compared the efficacy of baclofen, up to 180 mg/day (in three daily administrations), to placebo in 310 AUD patients for 26 weeks [(44); Table 1]. This RCT found no difference between baclofen and placebo in the percentage of abstinent patients and in the reduction of alcohol consumption. Compared to the study of Beraha et al. (43), this RCT recruited a similar percentage of women (27%), participants were abstinent for a similar period of time prior to the start of the study medication (3–14 days) and consumed a slightly lower amount of alcohol at baseline (95.5 g of alcohol per day for patients allocated to baclofen group, equal to 7.9 drinks per drinking day when 1 drink = 12 g of alcohol). However, the two RCTs differed in the duration, mean actual baclofen dose, presence of comorbid mental disorders, setting, and frequency of psychosocial treatment. The duration of the Reynaud et al. (44) RCT was longer than in the Beraha et al. (43) study (26 vs. 16 weeks). In this RCT, a higher percentage of participants reached the maximum dose of baclofen (66 vs. 16%) and participants received a higher mean daily dose of baclofen (153.5 vs. 93.6 mg). Both RCTs excluded participants with current severe mental disorders. However, participants with bipolar disorder were excluded in Reynaud et al. (44) and included by Beraha et al. (43). In the Reynaud et al. (44) RCT, all patients were seen as outpatients, whereas in the Beraha et al. (43) study 79% of participants were treated as in patients for the first 4–6 weeks. Participants also received psychotherapy sessions less frequently (every 2 weeks vs. weekly in the other RCT) and the placebo effect was lower (e.g., 11 vs. 66%).

Only one RCT using doses of baclofen up to 270 mg/day has been published [(45); Table 1]. The results of a second RCT (using up to 300 mg/day) have been presented at scientific meetings but have not yet been published in full (66). Unlike the other two similar RCTs presented above (43, 44), this study found that baclofen substantially increased the percentage of abstinent patients and cumulative abstinence duration compared to placebo [(45); see Table 1].

In this RCT (45), patients allocated to baclofen received a mean dose of 180 mg/day (in three daily administrations) (compared to 153.5 and 93.6 mg/day in the other 2 RCTs), and 36% of these patients achieved the maximum dose (vs. 66 and 16% in the other two RCTs with doses >100 mg/day). This RCT was conducted at a single outpatient unit and recruited 56 AUD participants with high baseline levels of alcohol consumption (206.2 g per day, equal to about 17 drinks per drinking day with 1 drink = 12 g of alcohol vs. 12 and 8 drinks per drinking day in the other 2 RCTs with doses >100 mg/day). The 3 RCTs did not differ in other characteristics. This RCT included 17 women (30%) and participants did not suffer from current severe mental disorders. The study lasted 24 weeks, participants were abstinent for a mean of ~12 days at the beginning of the trial and received supportive therapy (Medical Management).

#### Meta-Analyses

To date, there have been four meta-analyses of baclofen for the treatment of AUD, based on the studies described above [(67–70); see Table 2]. These meta-analyses vary in the number of RCTs evaluated between five (68) and 14 (67), as well as in the outcomes investigated. The most inclusive study (67) evaluated the efficacy of baclofen pooling the outcomes chosen by each single study as the primary outcome, and in two subgroups of outcomes, one related to abstinence and one to alcohol consumption. According to the results of this meta-analysis, baclofen did not differ significantly from placebo in any of the outcomes investigated.

**Table 2:** Meta-analyses.

	**Lesouef et al. (68)**	**Bschor et al. (67)**	**Pierce et al. (69)**	**Rose and Jones (70)**
**RCTs**				
Addolorato et al. (33)	X	X	X	X
Addolorato et al. (35)	X	X	X	X
Beraha et al. (43)		X	X	X
Garbutt et al. (34)	X	X	X	X
Garbutt et al. (34)	X	X		
Hauser et al. (36)		X	X	X
Jaury (66)		X	X	
Krupitsky et al. (71)				X
Krupitsky et al. (38)		X	X	X
Leggio et al. (42)		X	X	X
Mishra et al. (72)	X			
Morley et al. (40)		X	X	X
Morley et al. (41)		X	X	
Müller et al. (45)		X	X	X
Ponizovsky et al. (37)		X	X	X
Reynaud et al. (44)		X	X	X
Total number of studies	5	14	13	12
**NUMBER OF PARTICIPANTS**				
Baclofen	137	799	789	582
Placebo	135	723	713	543
Total participants	272	1,522	1502	1,125
**OUTCOMES EVALUATED**				
**Outcome selected by each study**	-	**X**	-	-
SMD	-	0.22	-	-
95% CI	-	−0.031–0.47	-	-
P	-	0.09	-	-
Heterogeneity	-	I^2^ = 75.2%	-	-
**% Abstinent participants**	X^*^	-	X^*^	X^*^
OR	2.79	-	1.93	2.67
95% CI	1.79–4.34	-	1.17–3.17	1.03–6.93
P	< 0.00001	-	0.01	0.04
Heterogeneity	I^2^ = 0%	-	I^2^ = 65%	I^2^ = 76%
**Abstinent days**	**X**	**X**	**X**	**X**
SMD	3.69	0.20	0.21	0.03
95% CI	−0.74–8.11	−0.08–0.49	−0.24–0.66	−0.10–0.15
P	0.10	0.16	0.37	0.67
Heterogeneity	I^2^ = 99%	I^2^ = 74.3%	I^2^ = 83%	I^2^ = 23%
**Drinking reduction**	-	**X**	-	-
SMD	-	0.28	-	-
95% CI	-	0.00–0.56	-	-
P	-	0.05	-	-
Heterogeneity	-	I^2^ = 71.9%	-	-
**Craving**	**X**	-	-	**X**
SMD	−1.6	-	-	−0.13
95% CI	−3.59–0.39	-	-	−0.36–0.09
P	0.12	-	-	0.24
Heterogeneity	I^2^ = 96%	-	-	I^2^ = 87%
**Time to lapse**	-	-	X^*^	-
SMD	-	-	0.42	-
95% CI	-	-	0.19–0.64	-
P	-	-	0.04	-
Heterogeneity	-	-	I^2^ = 60%	-
**Heavy drinking days**	-	-	-	**X**
SMD	-	-	-	−0.26
95% CI	-	-	-	−0.68–0.15
P	-	-	-	0.21
Heterogeneity	-	-	-	I^2^ = 95%
**Depression**	-	-	-	**X**
SMD	-	-	-	0.06
95% CI	-	-	-	−0.22–0.34
P	-	-	-	0.67
Heterogeneity	-	-	-	I^2^ = 87%
**Anxiety**	-	-	-	**X**
SMD	-	-	-	−0.03
95% CI	-	-	-	−0.24–0.18
P	-	-	-	0.77
Heterogeneity	-	-	-	I^2^ = 75%

* *The meta-analysis found a significant difference between baclofen and placebo. Assessment of heterogeneity: I^2^ > 50% = substantial level of heterogeneity. BAC, Baclofen; CI, confidence interval; OR, odd ratio; PLA, placebo; RCTs, randomized controlled trials; SMD, standardized mean difference*.

On the other hand, an earlier meta-analysis (68), including only baclofen studies with 30 mg/day baclofen, reported that baclofen significantly increased the rate of abstinent patients, compared to controls, at the end of treatment. A significant effect of baclofen for the same outcome was confirmed by two other recent meta-analyses in which more RCTs were included (69, 70). One of these meta-analyses also found that baclofen significantly increased the time to lapse, compared to placebo (69). However, a subgroup-analysis found a significant positive effect only across studies using 30–60 mg/day baclofen and not in the analysis of studies using higher doses of baclofen (69). Moreover, these meta-analyses did not find significant differences between baclofen and placebo on other important outcomes, such as the rate or number of abstinent days (67–70), alcohol craving (68, 70), depression (70), or anxiety (70). In one of the meta-analyses, the role of potential influencing factors was also explored (69) and found greater baclofen vs. placebo effect sizes in patients with higher baseline drinking levels.

#### Human Laboratory Studies

Human laboratory studies have investigated the effects of baclofen in experimental settings (73–75). One study investigated the safety of an acute administration of baclofen, in combination with alcohol, in 18 non-treatment seeking, heavy drinkers (defined as individuals who consumed a mean ≥28 drinks per week) (73). In this study, participants received three different doses of baclofen (0, 40, and 80 mg) and 0.75 g/kg of alcohol (about 4.5 standard drinks, with 1 standard drink = 12 grams of alcohol, in a man of 75 kg). The study found that both baclofen and alcohol impaired performance, but that few performance indicators were impaired to a greater extent when baclofen was combined with alcohol.

Another study found that 14 non-treatment seeking AUD subjects self-administered a lower amount of alcohol when they received 30 mg/day of baclofen compared to the sessions during which they received placebo. Furthermore, baclofen affected the biphasic effects of alcohol during the experimental alcohol administration session (74).

A more recent study by the same team investigated the effects induced by baclofen (30 mg/day) among a sample of 34 non-treatment seeking AUD individuals with high trait anxiety (75). They found that baclofen did not reduce the amount of alcohol consumed, but altered the subjective effects of alcohol, including an increase in the ratings of feeling high and intoxicated (75). Furthermore, in the same clinical study, they also found that baclofen may work by dissociating the link between an initial drink (priming) and subsequent alcohol consumption (self-administration) (76). Based on these results, the authors proposed that baclofen may act as a partial substitution AUD medication. A recent pharmaco-fMRI study found that baclofen specifically decreased alcohol cue-reactivity in brain areas involved in the processing of salient (appetitive and aversive) stimuli (77). However, the exact underlying biobehavioral mechanisms of baclofen in AUD individuals are still not completely understood (78–80).

### Possible Reasons For Inconsistent Results in Research to Date

The reasons for inter-study discrepancies are not fully understood. In general, it is well-established that clinical trials in AUD exhibit large variability because of a myriad of factors that affect outcome in AUD patients (46). In addition to the variability in doses (30–300 mg/day), studies varied in the following factors: age and gender; baseline severity of AUD and drinking levels; goal of the study (abstinence maintenance vs. reduced drinking); different cultures (with different drinking habits and genetic populations); addictive and psychiatric comorbidities; complications of AUD (such as cirrhosis or acquired brain injury); fixed or flexible dosing; individual adjustments; titration regimes; settings of the studies (inpatients, outpatients); completion of alcohol withdrawal and/or length of abstinence before treatment initiation; the intensity of concomitant psychological treatment and social support (leading to differences in the placebo effect); sample size; treatment duration; patient recruitment method; study endpoints; and prevalence of adverse events. In addition, it should be noted that in many studies only a minority of the patients received the intended or maximum allowed dose. Patients may require personalized doses, as some patients responded to 30 mg/day and achieved abstinence, while others required daily doses up to 300 mg/day. It has been observed that baclofen has a linear elimination in AUD patients, without saturation of baclofen clearance, over the range of doses usually administered to treat AUD [from 30 to 240 mg per day; 81, 82]. However, wide inter-individual variability of baclofen pharmacokinetics has been observed with highly different blood concentrations achieved by patients after the administration of the same dose (81). This may account for the differences in treatment response, where some patients, but not others, benefit from baclofen treatment. This pharmacokinetic variability may also be responsible for the wide range of doses required by different patients to achieve the desired effect. Furthermore, inter-study discrepancies may also be caused by differences in GABA-B receptor sensitivity (83).

Another issue requiring further investigation is the potential for a differential response by gender to baclofen treatment in terms of side-effects, safety, and tolerability. Among the RCTs published to date (see Table 1), one study did not report the gender of patients (38) and the others recruited a total of 302 female patients (25.3% of the entire sample) and 893 male patients (74.7%) (33–37, 39–45). Unfortunately, the individual RCTs did not provide data analyzed by gender and none of the meta-analyses (to date) have evaluated this aspect (67–70). The lack of gender analysis has been already described for the other medications approved for the treatment of AUD (84). Interestingly, an observational open-label, non-controlled study suggests that women may require significantly lower daily doses of baclofen than men (52). These preliminary findings suggest that the male to female ratio of patients in clinical trials may be an important factor in the overall efficacy, safety, and tolerability of baclofen in AUD patients, and requires further research.

### Baclofen for Alcohol Withdrawal Syndrome (AWS)

There is some preliminary evidence that baclofen may have a role as an adjuvant treatment of AWS (16). A number of case reports 85, 86, a retrospective chart review (87), and three small controlled studies (88–90) found that its administration reduced AWS severity. However, no study has been conducted to evaluate its potential effect in protection against seizures or Delirium Tremens (DTs). Accordingly, a recent meta-analysis concluded that there is insufficient evidence for recommending baclofen as a treatment for AWS (91). In summary, GABAergic medications like baclofen, and others, might play a beneficial adjuvant role managing AWS (92), however benzodiazepines remain the gold standard-of-care in AWS treatment, given that they are the only class of drugs with proven efficacy, not only in the treatment of AWS, but also in the prevention of AWS-related complications like seizures and DTs.

## Benefits And Challenges Of The Two Different Approaches Used To Administer Bacolofen: Fixed Dose vs. Flexible Dose

In clinical practice, baclofen is usually prescribed using either “fixed” or “flexible” doses. There are contrasting opinions on these two approaches. Therefore, both the *benefits* and c*hallenges* of “fixed doses” and “flexible doses” approaches of baclofen administration to treat AUD are described.

### Fixed Dose

Most of the RCTs started baclofen treatment with a daily dose of 5 mg, three times a day, gradually increased by 5–10 mg, every 3 days, up to a fixed dose of 30–80 mg/day. In response to side effects, baclofen administration was suspended or reduced. Because of its short half-life [2–6 h; 93], baclofen was administered in two, three, or four daily administrations.

#### Benefits

A recent meta-analysis found better results among studies using lower doses of baclofen compared to studies using higher doses [(69); see Table 2]. The use of lower doses is also associated with a lower risk of side-effects.

#### Challenges

Fixed maintenance doses are standard in RCTs and available evidence is driven by RCTs. However, fixed doses are rarely used in clinical practice (94). The optimal dose of baclofen varies substantially between patients, and treatment may be personalized through a slow increase of the dose. In addition, some patients may require a different distribution of daily administrations (e.g., late afternoon and early evening, instead of night time).

### Flexible Dose

This approach consists of progressively increasing the dose according to the balance of beneficial and unwanted effects. The dose required may vary widely from 30 mg/day up to more than 300 mg/day (some uncontrolled studies reported doses up to 560 mg/day), with baclofen prescribers using different titration regimes to increase the dose.

A common titration procedure is to increase the total daily dose of baclofen by one tablet of 10 mg every 3 days, or increasing each of the three daily doses by 5 mg every 3–7 days, until the treatment goal is reached. In case of significant side effects (e.g., severe sedation, dizziness, and/or confusion), the clinical advice is to stop increasing the dose or slow down the rate of increase: for example, 5 mg (half-tablet) rather than 10 mg increase every 3 days, 10 mg increase only every 4–7 days, or even less frequent dose increases.

#### Benefits

Some prescribers, in particular, those in France, claim that this titration method allows some patients to achieve a state of “indifference” toward alcohol, as initially described by Olivier Ameisen (47).

#### Challenges

There is a lack of clear evidence supporting this approach, as few studies have used it in a rigorous manner (95) and one of the meta-analyses has failed to show a significant effect of daily doses of baclofen >100 mg (69). Moreover, the use of higher doses of baclofen might be related to a higher risk of its relevant side-effects (96).

## Baclofen (Off-label) Use For The Treatment Of Moderate To Severe AUD

### General Considerations

As there is still debate about the efficacy of baclofen and how best to prescribe it, baclofen has been suggested to be prescribed only when approved pharmacological treatments have failed (2). However, in some countries, experienced prescribers may use it as a first line treatment in selected patients, such as those with liver disease, for whom other drugs may be contraindicated (97).

### Treatment Initiation

Baclofen treatment should always be initiated under careful medical oversight, by a prescriber with knowledge and training in this area. Evaluation of renal function is recommended before starting baclofen administration since renal insufficiency can be a cause of rapid accumulation of circulating baclofen, and may cause acute adverse events, particularly mental confusion (98).

### Patient Information

Patients should be clearly informed about the off-label use, potential benefits, side effects, and safety issues of this treatment, and the treatment plan, including who to contact in case of concerns. They should also receive comprehensive written information about baclofen treatment, including clear dosage regimes and a side effect profile. Documented, informed consent should be obtained from all patients. Patients with AUD may have mild cognitive deficits, potentially making it difficult for them to follow instructions. In these cases, when possible and with patient consent, it may be helpful if somebody close to the patient (spouse, relative, friend, or care worker) participates in the monitoring of the treatment.

### Goals of Treatment

Goals of treatment should be discussed and agreed with the patient. The patient needs to be aware that the effective dose to achieve his/her treatment goals may vary considerably. Patients should be informed that reaching the effective dose may be challenging, and that when doses are increased, baclofen may induce adverse effects, some of them potentially severe.

### Prior Detoxification or Initiation While Still Drinking

All the RCTs conducted to investigate baclofen efficacy recruited AUD patients who were active drinkers, and had stopped drinking prior to the start of the trial from 24 h (33) to 21 days (43, 45), with a mean of 12 days. In one RCT, most participants were treated as inpatients for the first 4–6 weeks (43). Whether alcohol detoxification is necessary prior to initiation of baclofen in clinical practice remains an open question. It is well-known that alcohol and baclofen have some side effects in common, and that the sedative effects of both drugs may potentiate each other (99). Accordingly, patients should be informed of the higher risk of sedation and overdose when taking baclofen while (still) drinking alcohol or using benzodiazepines (100).

### Safety Considerations and Specific Cautions

Some physicians choose to limit the dose in view of safety concerns around higher doses of baclofen. Other physicians increase the dose as high as needed, aiming for abstinence or low-risk drinking levels for active drinkers, or maintenance of abstinence for those who have already achieved it. These different options should be discussed with the patient. During the first visit, patients need to be informed that there are broadly two types of side effects: frequently occurring non-severe ones that are mainly benign and typically disappear spontaneously (or with dose reduction), and sporadically occurring dangerous side effects. The potentially dangerous side effects are seizures, respiratory depression with sleep apnea and potentially coma (in case of intoxication), severe mood disorders (mania or depression, with the risk of suicide), and mental confusion/delirium.

Driving a car, operating heavy machinery, working on scaffolding (for building workers), or using potentially dangerous tools (e.g., power tools), should be discouraged during the first weeks of treatment until patients learn how sedation affects them, and the treatment dose is stable. It is prudent to start and increase baclofen treatment on a non-working day, so the patient can assess the degree of sedative effect.

Patients should be advised to avoid drinking when they are taking baclofen, because of the risk of excessive sedation induced by the combination of the two substances (99). Patients should also be advised of the risk of overdose, if they take doses of baclofen higher than those prescribed (100). Finally, patients should be informed that baclofen treatment should be started and ended slowly, to reduce the risk of adverse events and withdrawal symptoms. Baclofen withdrawal syndrome might be associated with confusion, agitation, seizures, and delirium, and may be confused with AWS (101).

### Patients at Risk For Baclofen Overdose

Accidental and intentional baclofen overdose presents a particular challenge and may be fatal or lead to coma and seizures requiring prolonged intensive care treatment (96, 102, 103). It is noteworthy that calls to the National Poisons Centre of France have escalated during the past decade, i.e., the period when minimally supervised baclofen use for AUD increased (99, 100, 104). For instance, a retrospective study conducted in France found a progressive increase of baclofen overdoses among AUD patients between 2008 and 2013 (104). These cases comprised of 220 suicide attempts and 74 cases of unintentional intoxication, even if, in most of the cases, the suicide attempts were not directly attributable to baclofen itself. Therefore, patients at risk of overdose—including those with history of self-harm, over-dose, current suicidal ideation, or repeated and recent suicidal attempts—should not be prescribed baclofen. This risk can particularly pertain to patients with severe personality disorders, for example with borderline personality disorders, who are more likely to use baclofen for self-poisoning (97). However, it is not possible to exclude the role of alcohol consumption in some cases of baclofen overdose.

In some settings, controlled dispensing may be available, and while no such trials have been reported, this may allow for safer use of baclofen in a vulnerable patient population. Controlled dispensing may involve attending a pharmacy daily, or perhaps twice weekly, thus limiting patient access to medication for 1–3 days. A competent family member or other care-giver may undertake a similar role. Patients prescribed sedative medications (e.g., benzodiazepines, z-drugs, and antipsychotics) should be informed about the risk of excessive sedation, and respiratory depression in case of overdose, if baclofen is added to their therapy.

### Impairment of Renal Function

As noted previously, ~80% of baclofen is renally excreted, and thus baclofen may induce confusion, delirium, and other adverse effects in patients with renal failure (105, 106). Therefore, it is advisable to evaluate kidney function, checking for previous renal disease or current renal insufficiency, and requiring a renal function test.

### Use in Patients With Comorbid Conditions

#### Comorbid Psychiatric Conditions

The role of psychiatric comorbidity in explaining different responses to baclofen treatment, in terms of alcohol drinking outcomes, is still unclear (107, 108). However, as AUD patients often suffer from other mental disorders, potential baclofen effects on psychiatric comorbidity should be considered.

#### Bipolar Affective Disorder

About one third of patients with bipolar disorder have a comorbid AUD (109). Baclofen may elevate the patient's mood, inducing manic episodes (110). This mood elevation can also occur in patients with no known history of bipolar disorder, so a careful personal and familial history should be taken prior to starting baclofen treatment. Baclofen treatment of patients with known bipolar disorder require co-management with a psychiatrist. All patients should be warned about the risk of mood changes and told to discuss them with their treating doctor.

#### Anxiety

There is some suggestion that baclofen treatment may be effective in reducing comorbid anxiety symptoms in AUD patients (33, 34, 71). In one RCT, baclofen was more effective than placebo only in AUD patients with a comorbid anxiety disorder (40). However, the results of a recent meta-analysis did not support the hypothesis that baclofen treatment will also reduce anxiety symptoms (70).

#### Use of Baclofen in Patients Affected by Other Substance Use Disorders

Baclofen has also been used for the treatment of other substance use disorders (111). A few RCTs investigated its efficacy in the treatment of opioid withdrawal (112, 113), cocaine use disorder (114, 115), opioid use disorder (116), nicotine use disorder (42, 117), and methamphetamine use disorder (118). Some of these RCTs found positive results in favor of baclofen among patients with an opiate withdrawal syndrome (112, 113), and nicotine use disorders (42, 117). Notably, one of these studies found that 80 mg/day baclofen increased the rate of abstinent days from co-use of alcohol and tobacco in AUD and heavy-smoking individuals (42). However, other RCTs found no difference between baclofen and placebo in patients affected by cocaine use disorder (114), opioid use disorder (116), or methamphetamine use disorder (118). These inconsistent findings do not allow us to draw conclusions on baclofen efficacy in the treatment of substances use disorders other than alcohol. However, baclofen may be suggested for patients affected by AUD and comorbidity with other substance use disorders for which no approved drugs are available (111).

### Comorbid Physical Conditions

#### Liver Disease

The efficacy and safety of baclofen to facilitate maintenance of alcohol abstinence and prevention of relapse in AUD patients affected by liver cirrhosis (complicated or not with ascites) was first reported in an RCT by Addolorato et al. (35), in which a dose of 30 mg/day was utilized (119). These positive findings were then supported by retrospective studies (51, 55, 64, 65) and by one recent RCT (41), while another RCT in AUD patients with liver impairment did not report differences between baclofen and placebo (36), as detailed above. Baclofen treatment should be avoided in patients with liver cirrhosis complicated by encephalopathy (120) or administered at lower doses (e.g., 15 mg/day) among patients with hepatorenal syndrome (121). However, patients with these severe disorders rarely require pharmacological treatment to reduce or stop alcohol consumption given their already serious clinical condition.

#### Epilepsy

Baclofen lowers the seizure threshold and may precipitate seizures in people with a history of epilepsy. Therefore, it is essential to evaluate possible vulnerability to seizures. Epilepsy is a contraindication for the use of baclofen in some countries. Baclofen treatment in people with current epilepsy requires co-management with a neurologist.

#### Cardiovascular and Respiratory Diseases

Baclofen has infrequent, but well-established, effects on the cardiovascular and respiratory system, especially in overdose (104). It can slightly decrease blood pressure and heart rate, or cause hypertension, arrhythmia, and palpitations related to autonomic nervous system dysfunctions that are more likely linked with higher doses of baclofen (122). It can also potentiate the effect of antihypertensive drugs. Regarding the respiratory system, it can cause dyspnea and respiratory depression, and, most importantly, worsen obstructive sleep apnea (123). Baclofen has no substantial impact on cardiovascular and respiratory systems in healthy people, but physicians must be cautious in prescribing baclofen to patients with breathing and cardiovascular problems.

#### Parkinson's Disease

Baclofen can worsen the side effects of levodopa, possibly causing hallucinations, delusions, and confusion (124). However, a recent study found promising results using a combination of baclofen and acamprosate in a preclinical model of Parkinson's disease (125).

#### Urinary Incontinence

Urinary incontinence may be worsened by baclofen. Possible urinary incontinence should be investigated. Patients with this disorder may receive baclofen treatment, but the dose should be increased slowly.

#### Other Physical Disorders

Some studies reported that baclofen treatment was associated with sleep disturbance among AUD patients (44, 63). These findings are supported by preclinical evidence showing that baclofen may alter normal sleep patterns in animal models (126, 127). As AUD patients often suffer from disturbed sleep [particularly during AWS; APA (3)], it is possible that baclofen treatment may increase the risk and/or severity of sleep disorders among AUD patients. On the other hand, baclofen treatment has also been found to improve sleep among AUD patients, by helping them to achieve and maintain abstinence, or reducing alcohol consumption to low risk levels (128, 129).

Sporadic cases of sexual dysfunction have been reported among patients using baclofen to treat spasticity (130) and AUD (52, 63). As excessive alcohol consumption is a known cause of sexual dysfunction, baclofen treatment may worsen these disorders among AUD patients already suffering from sexual dysfunction. However, as with sleep disorders, it is possible that baclofen treatment, by helping AUD patients to achieve and maintain abstinence from alcohol or reducing alcohol consumption to low risk levels, may improve sexual function.

### Special Populations

#### Adolescence

No RCT has been conducted to investigate the effectiveness and safety of baclofen in adolescent patients with AUD. However, baclofen has been used in adolescents with severe spinal spasticity (17).

#### Pregnancy

Pregnant women with AUD raise genuine ethical dilemmas because of the potential risks to the fetus of using medications during pregnancy. As reliable studies are lacking, drug information agencies advise against the use of baclofen during pregnancy.

#### Elderly and Frail Patients

Baclofen can cause fatigue, sedation, and somnolence, which are often accompanied by decreased mobility and balance problems, especially in older people already suffering from these difficulties before starting baclofen treatment. Patients must be aware that these effects are usually tolerable, but that they may also be intense, with a risk of falls and falling asleep abruptly.

## Conclusions

Despite controversies regarding the efficacy and justification of the “off-label” use of baclofen treatment for patients with AUD, there is consensus that baclofen is a promising medication to treat moderate to severe AUD (2). Baclofen plays an important role in the clinical treatment of AUD patients in some European countries and Australia, particularly in patients who are not responsive to the available registered medications and/or in AUD patients with significant liver disease. However, in other countries (e.g., in the US), baclofen has a very low uptake for AUD treatment (131). As for the other drugs to treat AUD, there is no clear evidence on the ideal duration of treatment. Baclofen may be suggested to help patients with AUD to maintain abstinence, if they have already achieved it, or to achieve abstinence if they are still actively drinking. However, patients need to be advised of the potential high risk of sedation due to the combination of two different sedative drugs (i.e., alcohol and baclofen). Further studies are needed to evaluate the potential efficacy and safety of baclofen in different AUD patient groups (e.g., women, adolescents, the elderly, and during pregnancy), the ideal duration of treatment, as well as to clarify risks due to the combination of alcohol and baclofen.

## Author Contributions

RdB, PdL, PH, MH, PJ, and RA drafted the initial document. RdB, LL, JS, MH, and RA drafted the full-text manuscript and coordinated revisions before and after each round, up to completion of the manuscript and submission. All authors contributed to the manuscript and approved its final version.

### Conflict of Interest Statement

H-JA reports personal fees from Ethypharm, grants, personal fees and non-financial support from Lundbeck, personal fees and non-financial support from D&A Pharma, other from Pfizer, other from Lilly, other from Indivior, other from AbbVie, other from Arbor Pharmaceuticals, other from Alkermes, other from Amygdala Neurosciences, outside the submitted work. PJ reports personal fees from Polpharma, outside the submitted work. AL-H reports grants and personal fees from Lundbeck, personal fees from Silence Therapeutics, other from Opiant, other from Lightlake, other from Britannia, grants from GSK, personal fees from Janssen-Cilag, personal fees from Pfizer, personal fees from Sanofi-Aventis, during the conduct of the study. CM reports personal fees from Silence Therapeutics, outside the submitted work. BR reports personal fees from Ethypharm, outside the submitted work. WvdB reports personal fees from Lundbeck, personal fees from Eli Lilly, personal fees from Indivior, personal fees from Mundipharma, personal fees from Bioproject, personal fees from D&A Pharma, personal fees from Novartis, personal fees from Opiant Pharmaceuticals, outside the submitted work. The remaining authors declare that the research was conducted in the absence of any commercial or financial relationships that could be construed as a potential conflict of interest.
